# The arc of Buhler: special considerations when performing pancreaticoduodenectomy

**DOI:** 10.1186/s40792-016-0149-2

**Published:** 2016-03-07

**Authors:** Yumiko Kageyama, Takashi Kokudo, Katsumi Amikura, Yoshihiro Miyazaki, Amane Takahashi, Hirohiko Sakamoto

**Affiliations:** Division of Gastroenterological Surgery, Saitama Cancer Center, 780 Komuro, Ina-machi, Kita-adachi gun, Saitama, Japan

**Keywords:** Arc of Buhler, Pancreaticoduodenectomy, Celiac trunk, Stenosis

## Abstract

A 74-year-old female was diagnosed as having a carcinoma of the papilla of Vater. Preoperative computed tomography showed stenosis of the celiac trunk and an enlarged artery arising from the superior mesenteric artery (SMA) joining the root of the splenic artery. Since this artery communicated with the SMA and the celiac trunk, independently of the gastroduodenal and dorsal pancreatic arteries, it was considered to be the arc of Buhler (AOB). The arterial blood flow to the liver, spleen, and stomach appeared to depend on the AOB, such that AOB preservation was considered to be essential. A subtotal stomach-preserving pancreaticoduodenectomy with preservation of the AOB was thus performed. Although AOB is a relatively infrequent type of arterial communication between the SMA and the celiac trunk, it needs to be preserved during pancreaticoduodenectomy when celiac trunk stenosis is present.

## Background

The arc of Buhler (AOB) is a persistent embryonal ventral anastomosis between the superior mesenteric artery (SMA) and the celiac trunk [1, 2]. The most common and important anastomoses between the SMA and the celiac trunk are the gastroduodenal artery (GDA), the pancreaticoduodenal artery arcade, and the dorsal pancreatic artery (DPA). AOB is rarely reported [3]. Enlargement of and aneurysms involving these anastomoses are usually associated with stenosis or occlusion of the celiac trunk [3–6]. We herein report the first case, to our knowledge, undergoing pancreaticoduodenectomy in which preservation of the AOB was required.

## Case presentation

A 74-year-old previously healthy woman was referred to our hospital by a neighborhood doctor for detailed examination of asymptomatic elevation of liver enzymes and bile duct dilatation identified on abdominal ultrasonography. Laboratory examinations revealed elevated hepatic and biliary enzymes: total bilirubin 2.0 mg/dl (normal range, 0.1–1.2 mg/dl), aspartate amino-transferase135 IU/l (normal range, 5–30 IU/l), alanine amino-transferase127 IU/l (normal range, 3–35 IU/l), alkaline phosphatase 2284 IU/l (normal range, 90–300 IU/l), and γ-glutamyl transpeptidase 1435 IU/l (normal range, 1–28 IU/l). Levels of serum tumor markers, including carcinoembryonic antigen and carbohydrate antigen 19-9, were within normal limits.

**Fig. 1:**
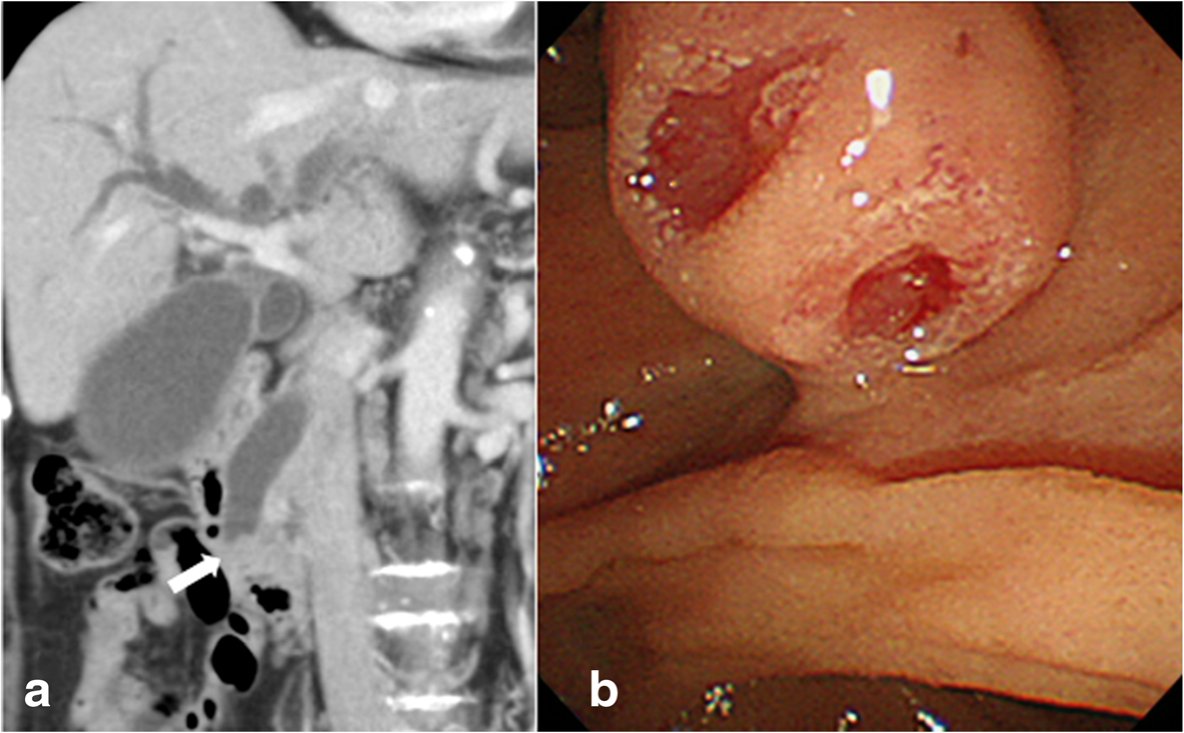
**a** Computed tomography showing the dilated common bile duct and the ampullary tumor *(arrow).* **b** The gastroscopic findings of the ampullary tumor

Contrast-enhanced abdominal computed tomography (CT) showed dilatation of the common bile duct and an ampullary tumor 12 mm in diameter with neither lymph node nor distant metastasis (Fig. 1a). Gastroscopy revealed an ampullary tumor with ulceration (Fig. 1b). Biopsy of the tumor yielded a pathological diagnosis of adenocarcinoma. Endoscopic retrograde cholangiopancreatography was performed, and a plastic stent was placed in the bile duct for biliary drainage.

**Fig. 2:**
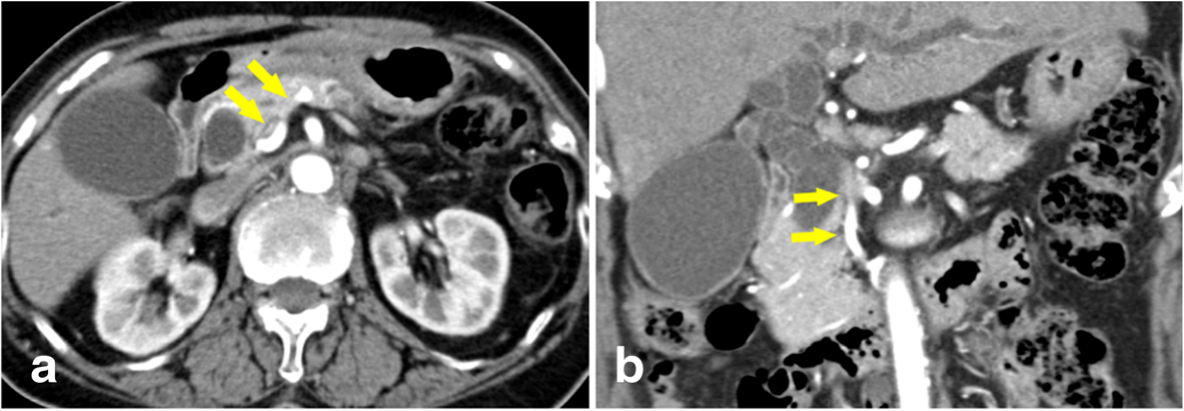
**a**, **b** Computed tomography image showing the arc of Buhler

**Fig. 3:**
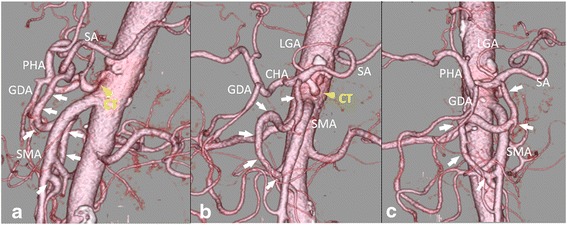
**a**–**c** A three-dimensional reconstructed computed tomography image showing the arc of Buhler. The *yellow arrowhead* indicates stenosis of the celiac trunk, and the *arrow* indicates the arc of Buhler. *CHA* common hepatic artery, *CT* celiac trunk, *GDA* gastroduodenal artery, *LGA* left gastric artery, *PHA* proper hepatic artery, *SA* splenic artery, *SMA* superior mesenteric artery

CT showed stenosis of the celiac trunk and an enlarged artery arising from the SMA joining the root of the splenic artery, which ran just behind the portal vein (Figs. 2 and 3). There was no evidence of midline bow-formed ligament sclerosis and since calcification and stenosis of other arteries were present, the celiac trunk stenosis was attributed to arteriosclerosis. Since this artery communicated with the SMA and the celiac trunk, independently of the GDA and the DPA, it was considered to be the AOB. The arterial blood flow to the liver, spleen, and stomach appeared to depend on the AOB. We, therefore, decided to give priority to preserving the AOB over a radical lymphadenectomy.

**Fig. 4:**
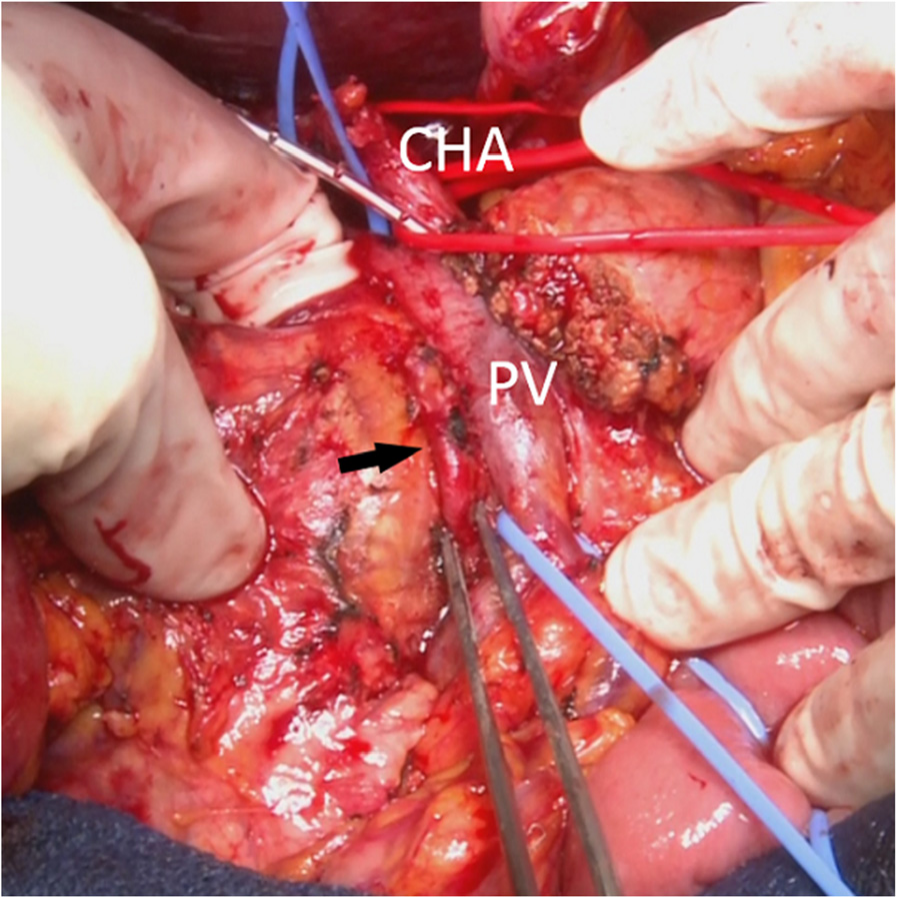
Intraoperative identification of the arc of Buhler (*arrow*)

**Fig. 5:**
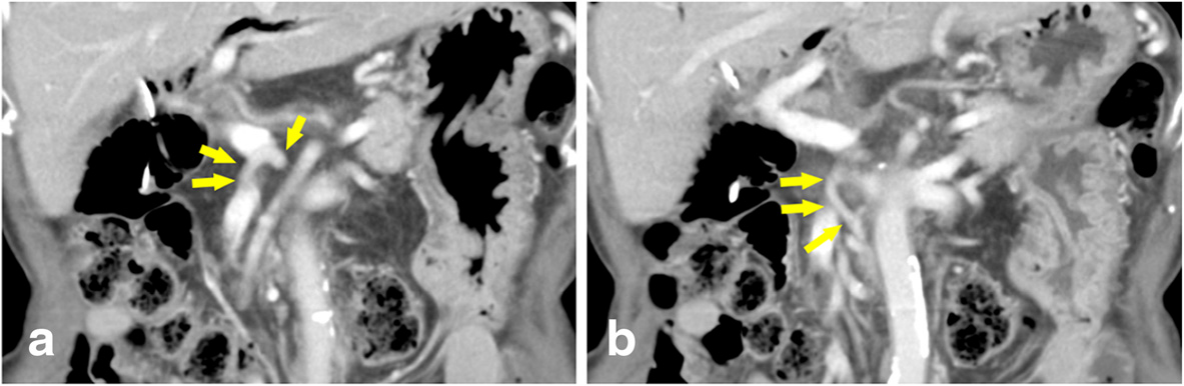
**a**, **b** Computed tomography image obtained 3 months postoperatively showing patency of the arc of Buhler

The patient was diagnosed as having an adenocarcinoma of the ampulla of Vater without lymph node or distant metastasis. We performed a subtotal stomach-preserving pancreaticoduodenectomy with regional lymph node dissection. After dissection of the pancreas and duodenum, we first completely dissected the pancreatic head from the portal vein. We then identified the AOB adjacent to the SMA (Fig. 4). Since the AOB ran just beside the SMA and the two arteries were covered by a continuous perivascular nerve plexus, dissection to separate these two arteries was not performed to avoid accidental injury of the AOB. Therefore, we set the dissection line outside the AOB and SMA in order to preserve the AOB and dissected the tissue between the uncinate process of the pancreas and these two arteries. Pathological examination revealed an ampullary adenocarcinoma without lymph node metastasis. The postoperative course was uneventful, and the patient was discharged from our hospital on postoperative day 14. The CT scan performed 3 months after the operation confirmed patency of the AOB (Fig. 5).

### Discussion

Preoperative recognition of arterial anomalies is important during pancreaticoduodenectomy [3, 7]. The AOB was first described by Buhler and Tandler in 1904 [1, 2]. This anastomotic artery is independent of the GDA and the DPA. The SMA and the celiac trunk arise during the prenatal period from the 10th and 13th segmental arteries, respectively, which arise from the aorta. These segmental arteries are connected by a ventral anastomosis, which usually regresses. Failure of this regression leads to persistence of the ventral communication between the SMA and the celiac trunk or one of its branches, the so-called the AOB. Previous studies found AOB prevalence to range from 1 to 4 % [3, 8, 9].

We conducted a literature review including the Japanese literature which identified 12 reports describing AOB, using PubMed with the key word “arc of Buhler” (Table 1). Most of these reports were about aneurysms of the AOB due to celiac trunk stenosis. Our literature review yielded 30 AOB cases, 7 with celiac trunk stenosis or occlusion, 14 with celiac trunk and/or SMA stenosis or occlusion, and 9 without arterial stenosis. The arterial network surrounding the pancreas is an important source of collateral blood supply in cases with stenosis or occlusion of the celiac trunk, SMA, or splenic arteries [9, 10]. There are several strategies for managing hepatic blood flow during pancreaticoduodenectomy in celiac artery compression syndrome, such as stent placement or laparoscopic median arcuate ligament release [11, 12]. In cases with celiac artery compression syndrome, hepatic blood supply depends on the GDA, such that intraoperative division of the GDA results in ischemia of the liver. In our case, the blood supply depended on not only the GDA but also, in fact mainly, on the enlarged AOB. Therefore, the collateral blood supply from the SMA to the liver was not interrupted. On the other hand, since the arterial flow in the hepatic artery can be maintained via direct flow from the celiac trunk, it is not necessary to preserve the AOB when stenosis or occlusion of the celiac trunk is not present. This is the first report, to our knowledge, describing a case undergoing pancreaticoduodenectomy in which preservation of the AOB was required because of celiac trunk stenosis.

**Table 1:** Previous reports on the arc of Buhler

Author (year)	Disease		Treatment
Buhler (1904) [1]	n.a.	*n* = 1	n.a.
Grabbe (1980) [8]	n.a.	*n* = 14	n.a.
Kugai (1996) [13]	AOB aneurysm	*n* = 1	Resection
Myers (1998) [14]	AOB aneurysm	*n* = 1	Resection, vein grafting
McNulty (2001) [3]	Gastrointestinal hemorrhage	*n* = 3	n.a.
Tokura (2002) [15]	PDA aneurysm	*n* = 1	TAE
Saad (2005) [9]	Liver transplant donors	*n* = 4	n.a.
Dubel (2007) [4]	AOB aneurysm	*n* = 1	TAE
Jeong (2008) [5]	AOB aneurysm	*n* = 1	TAE
Kallamadi (2009) [6]	Mesenteric ischemia	*n* = 1	n.a.
Jayia (2011) [16]	AOB aneurysm	*n* = 1	TAE
Rusu (2011) [17]	n.a.	*n* = 1	n.a.
Our case	Ampullary carcinoma	*n* = 1	Pancreaticoduodenectomy, preserving AOB

*AOB* arc of Buhler, *PDA* pancreaticoduodenal artery, *TAE* transcatheter embolization, *n.a*. not available

## Conclusions

When celiac trunk stenosis or obstruction exists, the blood flow in the hepatic artery depends on the communicating arteries between the celiac trunk and the SMA. Although AOB is a relatively infrequent type of arterial communication, our present case underscores the importance of identifying and preserving this arterial anomaly when performing pancreaticoduodenectomy.

## Consent

Written informed consent was obtained from the patient for the publication of this case report and any accompanying images.

## Abbreviations

AOB: arc of Buhler
CT: computed tomography
DPA: dorsal pancreatic artery
GDA: gastroduodenal artery
SMA: superior mesenteric artery
